# Addressing a gap in community-engaged research: reporting results of congregations’ implementation capacity assessment to research participants

**DOI:** 10.3389/fpubh.2026.1826775

**Published:** 2026-05-28

**Authors:** Jemal Gishe, Rebecca Selove, Sanjoy Saha, Thomas J. Waltz, Korab Idrizi, Abdulrahman Sankoh, Melissa Arismendy, Kristin Clarkson, Neely Williams, Omaràn D. Lee, Sharon Jones, Leah Alexander, David G. Schlundt

**Affiliations:** 1 Tennessee State University, Nashville, TN, United States; 2 Eastern Michigan University, Ypsilanti, MI, United States; 3 Congregational Health and Education Network, Nashville, TN, United States; 4 Tennessee Community Engagement Alliance (TN CEAL), Nashville, TN, United States; 5 SíCare Powered by, Reach One Teach One Foundation Inc, Nashville, TN, United States; 6 Vanderbilt University, Nashville, TN, United States; 7 Meharry Medical College, Nashville, TN, United States

**Keywords:** CFIR, community-engaged research, ERIC, faith-based organizations, implementation science, tailored feedback

## Abstract

**Background:**

Community-engaged research depends on sharing findings effectively; however, few tools translate implementation capacity and readiness data into actionable feedback. This gap limits community organizations’ ability to use results to guide health program implementation, build trust in research, and sustain ongoing participation. This study developed and used a tailored survey response report process for congregations participating in the Engaging Partners in Caring Communities (EPICC) project to support implementation planning and technical assistance.

**Methods:**

The EPICC CFIR-informed survey, grounded in the Consolidated Framework for Implementation Research (CFIR), was completed by teams from 30 congregations after they selected a health promotion evidence-based program. A multi-step process, informed by the Expert Recommendations for Implementing Change (ERIC), was developed to generate congregation-specific feedback reports. Satisfaction with the reports was assessed using responses from 80 participants.

**Results:**

Congregations varied widely in size and implementation capacity. Cost, design quality, and readiness for implementation were identified as the most common challenges. Tailored strength-based survey response reports were generated for all congregations. Satisfaction was high, with over 95% of respondents rating the reports as accurate, helpful, and satisfactory or better in reflecting their congregation and guiding implementation planning.

**Conclusion:**

A theory-based, strength-focused CFIR-ERIC assessment and feedback process was successfully adapted for faith-based congregations, extending implementation frameworks beyond healthcare settings. Tailored reports and interactive feedback were highly acceptable and provided actionable guidance for readiness and program implementation. This scalable approach may facilitate translation of implementation science to diverse community settings, although effects on implementation outcomes require further study.

## 1 Background

Community-engaged research (CEnR) has long been considered essential as an approach for effectively promoting health and reducing prevalence and impact of chronic diseases (1–3). Approaches to CEnR vary in the ways in which community members and partner organizations are involved in identifying research questions, selecting research methods, and managing the project (4). The value of community participation includes the goal of conducting research that is relevant and useful to the community, with greater potential for their health benefit (3, 5).

Researchers are ethically obligated to disseminate information obtained in CEnR to community members, including data summaries and conclusions (6, 7). The dissemination of these findings back to community members reflects a core principle of rigorous scientific practice, as results are more likely to be valid and have contextual relevance when community members are actively engaged in data interpretation (8). Community members are more likely to trust research findings when they have opportunities to review results and are more likely to continue participating in and contributing to research when its usefulness and relevance are made clear (9, 10).

Despite these CEnR principles, there is limited guidance on how to systematically return complex research findings, specifically implementation-related assessments to community partners in ways that are both interpretable and actionable. Although prior studies have applied frameworks such as Consolidated Framework for Implementation Research (CFIR) to engage non-academic organizations, these efforts have primarily focused on data collection and strategy selection rather than translating findings into tailored, usable feedback for community stakeholders (11–13).

Congregations serve as trusted community institutions, making them ideal settings for community-engaged research aimed at promoting health and reducing health disparities (14). This manuscript describes the process used to develop a tailored report that was presented to members of congregational teams who completed a CFIR-informed survey for their congregation. As part of EPICC (Engaging Partners in Caring Communities), an NIH-funded CEnR project, congregations in Nashville, TN were invited to select an evidence-based program (EBP) from six identified by the research team (15). Then the congregation’s implementation team completed one online CFIR-informed survey as a group. The research team then developed a tailored report of their survey responses that was presented to members of each congregational team for their review and feedback to the research team.

There have been calls for providing feedback to community-engaged partners (16), and some examples of feedback methods (11). However, few publications describe the development of a process for reporting the results of CEnR back to community participants (11).

This paper addresses that gap by describing the development and implementation of a theory-driven integrated strategy for returning CFIR-based implementation assessment results to community partners in a faith-based organizational context. The strategy includes summarizing the CFIR-informed survey, delivering the EPICC survey response report, and conducting a feedback session, all designed to provide tailored information on congregational strengths and challenges and to facilitate interpretation, engagement, and practical application relevant to the church’s capacity to implement an evidence-based health promotion program (EBP).

## 2 Methods

### 2.1 Survey development

Early in the survey development process, we conducted cognitive interviews with four pastors who completed the first iteration of the CFIR-informed survey. Items were adapted from other CFIR-informed surveys and the CFIR Wiki-page, described in a manuscript that is in development. Based on their feedback, several CFIR constructs were not included in the final instrument. Specifically, Trialability was omitted from the Intervention Characteristics domain; External Policy and Incentives was omitted from the Outer Setting domain; Other Personal Attributes was omitted from the Characteristics of Individuals domain; and Engaging, Formally Appointed Internal Implementation Leaders, External Change Agents, and Reflecting and Evaluating were omitted from the Process domain. No constructs were omitted from the Inner Setting domain. The final EPICC CFIR-informed survey consists of 140 items, of which 100 were directly mapped to 31 CFIR constructs across five domains (Table 1).

**Table 1:** CFIR domains and constructs used in CFIR-informed survey with brief descriptions.

CFIR domains	CFIR constructs	Brief description (# of CFIR items^1^)
Intervention characteristics	Intervention source	Importance of who developed the EBP (3)
	Evidence strength and quality	Importance of evidence of EBP benefits (1)
	Relative advantage	More effective than other programs church has offered (1)
	Adaptability	Can be adapted to fit congregation needs (1)
	Complexity	Requires changes in routines, easy to understand and deliver, fits in with long-range goals (4)
	Design quality and packaging	Reflects religious beliefs, cultural/ethnic identity (2)
	Cost	Cost of training, materials, time (3)
Outer setting	Patient needs and resources	Surveyed members about health topics of interest, EBP will meet their needs, will members face barriers to participation (3)
	Cosmopolitanism	Connection to larger groups of congregations that provide support for offering EBP (2)
	Peer pressure	Team is aware of health promotion programs in other congregations, members will value EBP more than one offered elsewhere (2)
Inner setting	Structural characteristics	Presence of health ministry or health team (2)
	Networks and communications	Communication modes used for communicating and delivering health-related messages to members (18)
	Culture	Ways church currently addresses health education and promotion through church activities and structure (10)
	Implementation climate	Acceptability of and support for a new health program (3)
	Tension for change	Interest in starting health ministry or new programs (2)
	Compatibility	History of offering, participating in, and documenting health and other church activities (8)
	Organizational incentives and rewards	History of recognition, rewards, and benefits related to health-related and other programs (5)
	Goals and feedback	Monitoring progress toward goals (2)
	Learning climate	Sense of responsibility and interest in new programming (2)
	Readiness for implementation	Training, space for, and experience with health promotion programs (7)
	Leadership engagement	Church leaders/clergy support in and involvement with health promotion (5)
	Available resources	Building is adequate for current programs (1)
	Access to knowledge and information	Leaders or staff receive information about health programs for congregations (1)
Characteristics of individuals	Knowledge and beliefs about the intervention	Leaders are informed about effectiveness of health promotion programs for organizations (1)
	Self-efficacy	Confidence that members will benefit from EBP (2)
	Individual stage of change	Experience with and readiness for offering a health promotion program (2)
	Individual identification with organization	Attitudes of implementation team members regarding importance and benefits of offering health promotion program (3)
Process	Planning	How likely are leaders to develop guidance for implementing new program (1)
	Opinion leaders	Active involvement of leaders in health promotion (1)
	Champions	At least one congregation member who will champion health promotion program (1)
	Fidelity	If EBP is offered, it will be offered as it was designed (1)

1 The number of individual CFIR items included in each construct.

The CFIR-informed survey constructs were used to assess each congregation’s capacity to implement and sustain EBPs. The remaining survey items captured key demographic and contextual information (e.g., space, church size), providing a richer understanding of the organizational environment. Each CFIR construct was evaluated for its potential to function as either a strength or a challenge to EBP implementation. To promote comprehensive and accurate reporting, the survey was completed collaboratively by the whole congregational implementation team, composed of 3–5 members. Group completion allowed participants to cross-validate responses and address knowledge gaps, as different team members might hold information about distinct organizational processes and resources.

### 2.2 Survey administration and summary

Once all members of a congregation’s team signed informed consent documents, they were asked to select one evidence-based program (EBP) from a list of resources carefully identified and selected by the EPICC team: Body and Soul; Building Healthy Families; Faith, Activity and Nutrition; Walk Your Heart to Health; Weight Wise; and We Can (15). Brief descriptions of each EBP were provided to the congregation, including a description of how the programs were chosen. Church teams were informed that all programs met criteria important to churches, including that they address health conditions disproportionately affecting people of color, include guidance for non-health professionals to implement the programs, and are free. The survey included items assessing the importance of these factors to the church team; however, the programs were selected to ensure that these characteristics of interventions would not present barriers to implementation. The EPICC website featured a five-step questionnaire designed to assist congregational teams in identifying the program that best fits their specific congregation, thereby facilitating informed decision-making.

After a congregation selected an EBP, the CFIR-informed survey link was emailed to the congregation’s team leader. The CFIR-informed survey is a web-based implementation capacity assessment tool developed by the EPICC research team using CFIR as its theoretical foundation (17). CFIR provides a structured approach for assessing factors that influence implementation outcomes, encompassing constructs across domains such as intervention characteristics, outer setting, inner setting, individual characteristics, and the implementation process (17, 18). Survey responses were collected through the secure Research Electronic Data Capture (REDCap) platform.

Upon completion, the survey data were downloaded from REDCap by the EPICC research team and imported into a custom-built Excel macro developed specifically for analyzing CFIR data. Survey responses were rated on a Likert scale ranging from 1 (lowest) to 4 (highest). For constructs assessed by multiple items, the median score was used to determine the final construct rating. Constructs with scores of 1 or 2 were categorized as challenges to implementation, while those with scores of 3 or 4 were considered strengths (17).

The macro included an adapted CFIR-ERIC Matching Tool (19). This tool linked identified CFIR challenges with relevant strategies from the Expert Recommendations for Implementing Change (ERIC) (20). The ERIC framework provides a comprehensive list of implementation strategies widely recognized in healthcare and public health contexts (19). However, the original language of ERIC strategies was designed primarily for clinical settings. In response, the EPICC research team collaborated with Dr. Thomas Waltz, the lead developer of the CFIR-ERIC Tool, and two church leaders who were part of the research team, to adapt the language and framing of ERIC strategies for faith-based organizations (FBOs). These adaptations ensured that the strategies were contextually relevant and practically applicable to congregational settings (21).

The Excel macro processed the survey data to generate a detailed summary of findings, including identified challenges and strengths; a subset of key strengths (i.e., constructs rated 4); and a prioritized list of ERIC strategies based on cumulative scores matched to the specific challenges reported by the congregation. These outputs were synthesized into a CFIR-ERIC matrix which, along with the raw survey data, was reviewed by the research team. Based on this analysis, the team prepared a strength-based preliminary draft of a congregation-specific survey response report, adapted from Holt et al. (22), which emphasized the importance of focusing on a congregation’s strengths.

The report included the highest-scoring strategy for each CFIR construct identified as a potential challenge, keeping each church’s report to no more than eight pages. Community advisors indicated this length would be helpful rather than overwhelming. When appropriate, complementary strategies were combined and described succinctly.

### 2.3 Steps in developing and formatting survey response report

**Figure 1:**
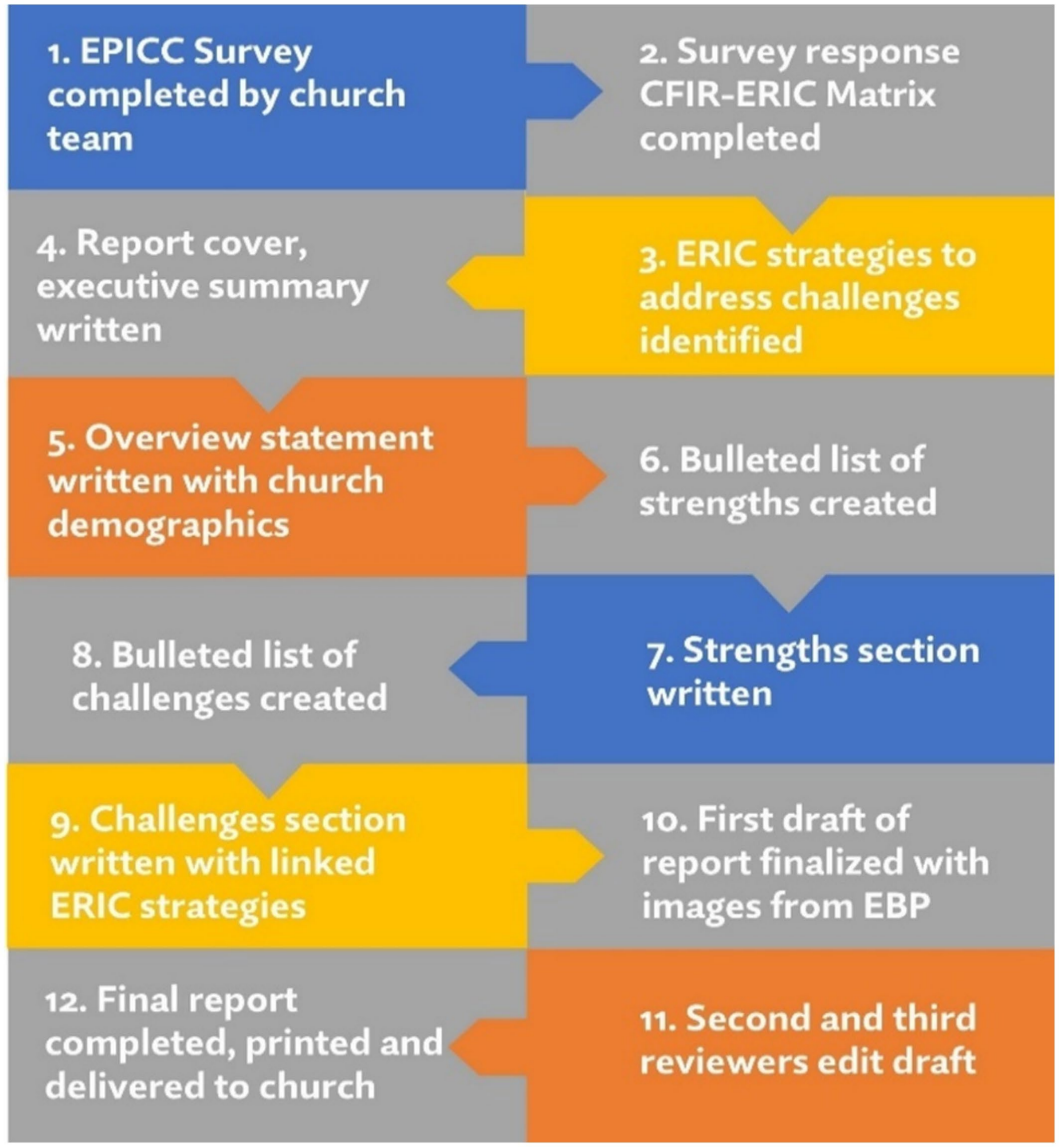
Step-by-step process for building the EPICC survey response report.

A standardized, multistep process was developed by the research team and used to develop individualized survey response reports for each participating congregation (see Figure 1). Each report began with a title page and executive summary identifying the congregation, survey completion date, and selected evidence-based program, followed by an overview describing the congregation’s pastor, team members, and contextual characteristics. An initial overview section highlighted four to five major strengths identified from high survey strength scores. Separate sections were created to present strengths and challenges which we called “areas for growth”, with strengths identified using high-scoring survey domains and for challenges identified using low-scoring domains across CFIR constructs. Standardized text templates were used to ensure consistency when incorporating descriptions of strengths and challenges.

For each CFIR construct identified as a challenge, corresponding ERIC implementation strategies were matched using strategy scores, and the highest-scoring strategy was selected and incorporated into the report. Reports were refined for clarity, conciseness, and flow, and strategy descriptions were enhanced using the EPICC ERIC Strategy Glossary (20) and evidence-based practice implementation resources. Final steps included formatting checks, page numbering, and the inclusion of images related to the congregation’s selected evidence-based program.

**Figure 2:**
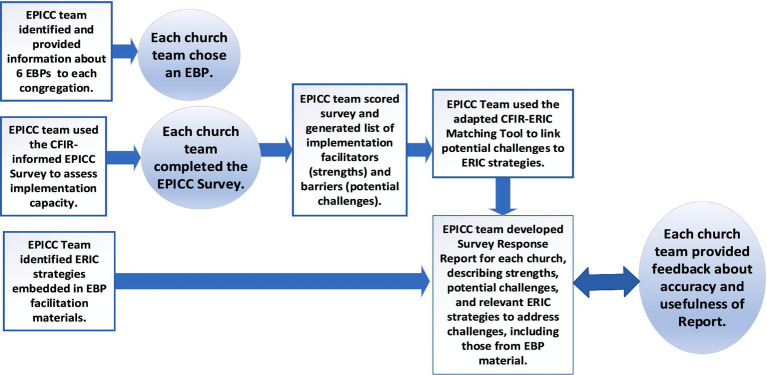
Steps involved in developing tailored survey response reports.

After congregations received the printed Survey Response Report and participated in a follow-up interview (will be included in another manuscript currently under development) with the implementation team, the congregation’s team also completed a multiple-choice questionnaire assessing their satisfaction with the report (Figure 2).

### 2.4 IRB approval and survey participants

The study protocol was approved by the Institutional Review Boards (IRBs) of Tennessee State University, and Vanderbilt University. Each church team’s member signed an informed consent document.

### 2.5 Data analysis

Descriptive statistics were computed to summarize study variables, including frequencies and percentages for categorical variables and medians with interquartile ranges (IQRs) and means with standard deviations (SDs) for ordinal variables. A Pearson correlation coefficient was calculated to assess the association between congregation size and implementation capacity. A normal distribution-based classification approach was used to categorize each congregation’s implementation capacity as beginner, intermediate, or advanced. All statistical analyses were conducted using SPSS Statistics for Windows (Version 29.0; IBM Corp., 2023).

## 3 Results

### 3.1 Demographic characteristics

A total of 30 congregations completed the EPICC survey, with one response submitted per congregation. In addition, 80 participants completed a congregational satisfaction questionnaire evaluating the survey response report. The questionnaire accompanied the printed reports as a hard-copy, multiple-choice instrument designed to assess satisfaction with the report. Each congregation received a sufficient number of paper questionnaires, along with stamped, pre-addressed envelopes for return to the study team, for all congregation team members identified as contributors to the survey response. Responses were received from 18 congregations, with the number of questionnaires returned per congregation ranging from 1 to 11. All data were entered by the research team into a Microsoft Excel file for analysis.

**Figure 3:**
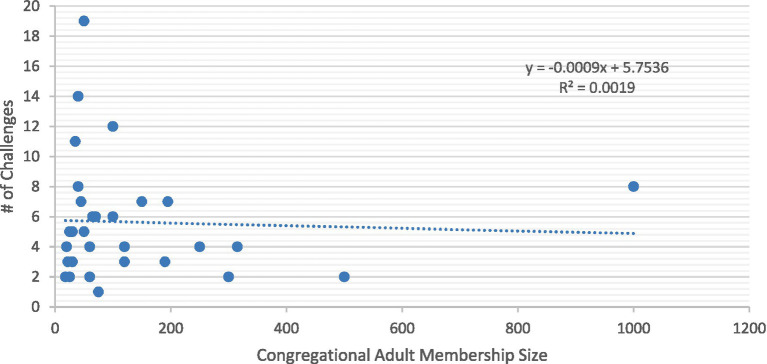
Scatterplots of the number of challenges by congregational adult membership size.

The demographic characteristics of the congregations demonstrated substantial variability in size and implementation capacity. Congregation size ranged from 18 to 1,000 adult members, with a median of 62.5 (IQR = 126.3) and a mean of 136.7 (SD = 196.9). No meaningful association was found between congregation size and the number of reported challenges (*r* = −0.04), indicating that implementation challenges occurred across congregations regardless of size (see Figure 3).

The most frequently reported challenges were cost (*n* = 28, 93.3% of the congregations), design quality and packaging (*n* = 24, 80% of the congregations), and readiness for implementation (*n* = 13, 43.3% of the congregations). Although cost was a challenge for most congregations, one of the criteria for selecting the EBPs by the EPICC Project was that there was no cost for training or materials.

The number of challenges per congregation ranged from 1 to 19 CFIR constructs, with a median of 4.5 and a mean of 5.6 (SD = 4) (see Figure 3). Based on the total number of challenges identified, four congregations were classified as beginner, 16 as intermediate, and 10 as advanced in implementation readiness, reflecting varying levels of implementation capacity across the sample.

In contrast, three CFIR constructs Intervention Source, Relative Advantage, and Complexity were identified as areas of strength by all 30 congregations. Additionally, 10 other CFIR constructs, including Evidence Strength and Quality, Adaptability, Implementation Climate, and Tension for Change, were identified as strengths by 29 of the 30 congregations (see Table 2).

**Table 2:** Descriptive statistics and strengths/challenges by CFIR construct across 30 congregations.

CFIR domains	CFIR constructs	Mean (S. D.)	Median (IQR)	# of Strengths (%)	# of Challenges (%)
Intervention characteristics	Intervention source	3.5 (0.51)	3 (1)	30 (100)	0 (0)
	Evidence strength and quality	3.7 (0.54)	4 (1)	29 (96.7)	1 (3.3)
	Relative advantage	3.8 (0.43)	4 (0)	30 (100)	0 (0)
	Adaptability	3.6 (0.56)	4 (1)	29 (96.7)	1 (3.3)
	Complexity	3.7 (0.46)	4 (1)	30 (100)	0 (0)
	Design quality and packaging	1.8 (0.93)	2 (1)	6 (20)	24 (80)
	Cost	1.3 (0.75)	1 (0)	2 (6.7)	28 (93.3)
Outer setting	Patient needs and resources	2.8 (0.8)	3 (1)	21(70)	9 (30)
	Cosmopolitanism	2.6 (1.23)	3 (3)	20 (66.7)	10 (33.3)
	Peer pressure	3.2 (0.93)	3 (2)	21 (70)	9 (30)
Inner setting	Structural characteristics	2.8 (1.37)	4 (3)	19 (63.3)	11 (36.7)
	Networks and communications	3.7 (0.68)	4 (0)	28 (93.3)	2 (6.7)
	Culture	2.8 (0.88)	3 (1)	20 (66.7)	10 (33.3)
	Implementation climate	3.6 (0.71)	4 (0.5)	29 (96.7)	1 (3.3)
	Tension for change	3.6 (0.56)	4 (1)	29(96.7)	1 (3.3)
	Compatibility	3.4 (0.88)	4 (1)	26 (86.7)	4 (13.3)
	Organizational incentives and rewards	2.9 (0.98)	3 (2)	21(70)	9 (30)
	Goals and feedback	2.8 (1.33)	3 (3)	20 (66.7)	10 (33.3)
	Learning climate	3.6 (0.56)	4 (1)	29 (96.7)	1 (3.3)
	Readiness for implementation	2.7 (1.42)	3 (3)	17 (56.7)	13 (43.3)
	Leadership engagement	3.6 (0.72)	4 (1)	29 (96.7)	1 (3.3)
	Available resources	3.8 (0.58)	4 (0)	29 (96.7)	1 (3.3)
	Access to knowledge and information	3.3 (0.91)	4 (1)	27 (90)	3 (10)
Characteristics of individuals	Knowledge and beliefs about the intervention	3.7 (0.53)	4 (0.5)	29 (96.7)	1 (3.3)
	Self-efficacy	3.6 (0.67)	4 (1)	29 (96.7)	1 (3.3)
	Individual stage of change	3.4 (0.67)	4 (1)	27 (90)	3 (10)
	Individual identification with organization	3.9 (0.4)	4 (0)	29 (96.7)	1 (3.3)
Process	Planning	3.6 (0.72)	4 (1)	28 (93.3)	2 (6.7)
	Opinion leaders	3.1 (1.02)	3 (1)	24 (80)	6 (20)
	Champions	3.5 (0.96)	4 (1)	26 (86.7)	4 (13.3)
	Fidelity	3.6 (0.75)	4 (0)	27 (90)	3 (10)

### 3.2 Congregational satisfaction with the survey response report

Overall, more than 95% of respondents reported being either satisfied or very satisfied across all items (see Table 3). Specifically, for the item “How satisfied were you with the printed Survey Response Report?”, 79.7% of respondents reported being very satisfied, 17.7% reported being satisfied, and only 2.5% reported being dissatisfied. Regarding the item “How accurately does the report describe your congregation?”, 65.0% indicated very accurate, 32.5% indicated accurate, and only 2.5% indicated inaccurate. Finally, for the item “How helpful is this report for expanding your health ministry?”, 84.4% of respondents indicated very helpful, 14.3% indicated helpful, and only 1.3% indicated rarely helpful.

**Table 3:** Congregational participants’ perceived satisfaction, accuracy, and helpfulness of the survey response report.

Survey items	Frequency (%)	Median, mean (SD)
How satisfied were you with the explanation you were given early on about the EPICC Project?		1, 1.2 (0.51)
Very Dissatisfied	1 (1.2)	
Dissatisfied	1 (1.2)	
Satisfied	11 (13.6)	
Very Satisfied	67 (83.8)	
How satisfied were you with the 6 options you had for a health promotion program?		1, 1.2 (0.42)
Satisfied	18 (22.5)	
Very Satisfied	62 (77.5)	
How satisfied were you with the information you had about each of the EBPs?		1, 1.9 (0.42)
Dissatisfied	1 (1.3)	
Satisfied	13 (16.3)	
Very Satisfied	66 (82.5)	
How satisfied were you with the information you were given about CHEN?		1, 1.2 (0.59)
Very Dissatisfied	1 (1.3)	
Dissatisfied	3 (3.8)	
Satisfied	11 (14.1)	
Very Satisfied	63 (80.8)	
How satisfied were you with the printed Survey Response Report?		1, 1.2 (0.479)
Dissatisfied	2 (2.5)	
Satisfied	14 (17.7)	
Very satisfied	63 (79.7)	
How accurately does the report describe your congregation?		1, 1.38 (0.537)
Inaccurate	2 (2.5)	
Accurate	26 (32.5)	
Very accurate	52 (65.0)	
How satisfied are you with the suggested strategies for addressing challenges your congregation might encounter?		1, 1.27 (0.548)
Very dissatisfied	1 (1.3)	
Dissatisfied	1 (1.3)	
Satisfied	16 (20.3)	
Very satisfied	61 (77.2)	
How helpful is this report for expanding your health ministry?		1, 1.17 (0.41)
Rarely helpful	1 (1.3)	
Helpful	11 (14.3)	
Very helpful	65 (84.4)	
How likely are you to participate in future EPICC activities?		1, 1.22 (0.503)
Unlikely	1 (1.3)	
Likely	14 (18.2)	
Very likely	62 (80.5)	
How likely are you to participate in CEnR projects in the future?		1, 1.27 (0.553)
Very unlikely	1 (1.3)	
Unlikely	1 (1.3)	
Likely	16 (20.8)	
Very likely	59 (76.6)	

## 4 Discussion

We described the development of a theory-based procedure to assess the strengths and challenges congregations may face when implementing EBPs through the EPICC project. Grounded in CFIR and ERIC frameworks, the research project guided congregational working groups to select one of six EBPs and complete a 140-item, CFIR-based REDCap survey collaboratively. Responses were scored using Excel macros that classify each construct as a strength or potential challenge, which were then mapped to ERIC strategies adapted for congregational settings. We found that the most reported challenges were cost, design quality and packaging, and readiness for implementation. Although the EBPs are available at no cost, implementing them may still require resources for materials such as printing, supplies, and food. Many congregations are small and operate with limited financial resources, making even modest implementation costs a potential barrier. In addition, congregations with limited experience in program implementation may feel hesitant to partner with a university to implement a complex evidence-based program. A qualitative study examining physical activity promotion in faith-based organizations, using a structured interview guide grounded in the CFIR framework, identified limited resources, church culture, prevailing norms, and competing priorities as key challenges to implementation (23). Our study moves beyond qualitatively identifying strengths and challenges across many congregations by providing a quantitative tool and algorithm for giving specific feedback to individual churches. In addition, the EPICC CFIR-informed survey and the Survey Response Report are designed to be used with a wide range of evidence-based programs and are not focused on a single physical activity intervention. In addition, the process proved workable across churches who had a wide range of readiness and capacity to plan and implement evidence-based programs.

Using a multi-stage process, summary data were prepared as a tailored report that was shared with the congregation’s working committee during a structured feedback session. Following these sessions, we collected satisfaction ratings to assess participants’ perceptions of the report and feedback process. A scoping review found that participants preferred receiving reports by mail and through face-to-face meetings rather than online methods (24). In this study, we likewise shared reports in person and provided guidance and training to support conducting face-to-face meetings within churches and left them with a detailed written report. The in-person delivery facilitated helping the implementation team read and understand the report, allowed for asking question, while the written report could easily be shared with other church leaders and members. The survey also showed high satisfaction with the written report.

Because CFIR and ERIC were originally developed for healthcare contexts (17, 21), we adapted constructs, refined terminology, and created a specific glossary to ensure relevance in faith-based organizations. We also developed a detailed algorithm to convert survey data into clear, contextually appropriate reports for congregational teams. Congregations reported high satisfaction, noting that findings were accurate and reflective of their settings. Feedback sessions with church teams supported shared interpretation, clarified questions, and initiated strategic planning. We described congregations along a readiness continuum from beginner to advanced and positioned the process as individualized, theory-based coaching. In providing the individualized survey response reports, we operationalized the ethical obligation to return research findings in a practical and actionable format.

The relational infrastructure of faith communities further supported acceptability. Trust shaped by pastoral leadership, shared mission, and longstanding community engagement influenced how feedback was received. Faith leaders acted as co-interpreters, reframing findings as collaborative capacity-building rather than external evaluation. This strengths-based, bidirectional model advanced power-sharing by engaging congregational teams in interpretation, planning, and adaptation, reinforcing ownership and self-efficacy. Clergy and lay leaders functioned not only as gatekeepers but as implementation partners with established communication networks and culturally grounded health messaging capacities.

This work contributes to implementation and dissemination science by demonstrating how established frameworks can be tailored for specialized community contexts. Beyond identifying site-specific strengths and challenges, the process generates EBP-specific strategy recommendations to guide rollout. The interactive feedback sessions and written reports provide tangible tools to support movement from readiness to planning to action. The approach offers a roadmap for adapting implementation science to other community settings, such as schools, childcare centers, senior centers, businesses, and parks and recreation organizations.

Limitations include geographic concentration in Nashville, Tennessee, potentially limiting generalizability. The faith-based focus may not translate directly to other settings without additional adaptation. The 140-item survey may be burdensome, and dichotomizing scores (1–2 as challenges; 3–4 as strengths) may obscure nuance. Although we collected satisfaction data from the working groups regarding the feedback reports, we have not yet presented evidence demonstrating that this approach improves satisfaction with the broader coaching process or leads to successful implementation of the EBPs.

## 5 Conclusion

Within the context of the EPICC project, we demonstrated that the CFIR and ERIC frameworks can be adapted for faith-based settings and used to create structured assessment and feedback processes that return findings to congregations through tailored written reports and interactive feedback sessions. Providing strength-based feedback with customized strategies to address challenges may enhance trust, increase readiness, and offer a practical roadmap for implementing and sustaining health behavior change. The full utility of this process remains to be established as we evaluate coaching outcomes and EBP implementation success.

Beyond EPICC, this study illustrates the feasibility of translating complex implementation frameworks into accessible, actionable tools for community institutions. Integrating structured assessment with relational facilitation highlights how scientific knowledge can become actionable within trusted community ecosystems. This approach is particularly relevant for addressing health disparities, as faith-based organizations often serve populations disproportionately affected by chronic disease and historically underrepresented in research. The relational infrastructure of congregations shaped by pastoral leadership, shared mission, and enduring community ties enhances the credibility and contextual relevance of implementation efforts. Engaging faith leaders as co-interpreters of ERIC strategies reframed feedback as collaborative capacity-building rather than external evaluation, underscoring the importance of relational trust in adapting implementation science to community and faith-based contexts.

## Data Availability

The raw data supporting the conclusions of this article will be made available by the authors, without undue reservation.

## References

[1] Wallerstein N Duran B Oetzel JG Minkler M. Community-based participatory research for health: Advancing social and health equity. 3rd ed. San Francisco, CA: Jossey-Bass (2023).

[2] Sanders Thompson VL Ackermann N Bauer KL Bowen DJ Goodman MS. Strategies of community engagement in research: definitions and classifications. Trans Behav Med. (2020) 11:441–51. doi: 10.1093/tbm/ibaa042, 32421173 PMC8135186

[3] Oetzel JG Wallerstein N Sanchez-Youngman S Boursaw B Dickson E Rivera J . Impact of participatory health research: a test of the community-based participatory research conceptual model. Biomed Res Int. (2018) 2018:7281405. doi: 10.1155/2018/7281405 29854784 PMC5941804

[4] Han HR Xu A Mendez KJW Okoye S Cudjoe J Bahouth M . Exploring community engaged research experiences and preferences: a multi-level qualitative investigation. Res Involv Engagem. (2021) 7:19. doi: 10.1186/s40900-021-00261-6, 33785074 PMC8008581

[5] Lachance L Coombe CM Brush BL Lee SY Jensen M Taffe B . Understanding the benefit–cost relationship in long-standing community-based participatory research (CBPR) partnerships: findings from the measurement approaches to partnership success (MAPS) study. J Appl Behav Sci. (2022) 58:513–36. doi: 10.1177/0021886320972193 36016649 PMC9398184

[6] Council for International Organizations of Medical Sciences. International Ethical Guidelines for Health-Related Research Involving Humans: Prepared by the Council for International Organizations of Medical Sciences (CIOMS) in Collaboration with the World Health Organization (WHO). Geneva: Council for International Organizations of Medical Sciences (2016). 40523065

[7] Villalobos A Blachman-Demner D Percy-Laurry A Belis D Bhattacharya M. Community and partner engagement in dissemination and implementation research at the National Institutes of Health: an analysis of recently funded studies and opportunities to advance the field. Implement Sci Commun. (2023) 4:77. doi: 10.1186/s43058-023-00462-y, 37438834 PMC10339604

[8] Wallerstein N. Engage for equity: advancing the fields of community-based participatory research and community-engaged research in community psychology and the social sciences. Am J Community Psychol. (2021) 67:251–5. doi: 10.1002/ajcp.12530, 34237169

[9] Cook S Mayers S Goggins K Schlundt D Bonnet K Williams N . Assessing research participant preferences for receiving study results. J Clin Transl Sci. (2020) 4:243–9. doi: 10.1017/cts.2020.25 PMC7348009 32695496

[10] Khodyakov D Williams P Bromley E Chung B Wells K. Using stakeholder input to inform an innovative research and policy initiative to improve depression in safety net communities. Prog Community Health Partnersh. (2017) 11:93–8. doi: 10.1353/cpr.2017.0012, 28529465 PMC5436044

[11] Palinkas LA Springgate B Cabassa LJ Shin M Garcia S Crabtree BF . Methods for community-engaged data collection and analysis in implementation research. Implement Sci Commun. (2025) 6:38. doi: 10.1186/s43058-025-00500-1 40197496 PMC11978136

[12] Lam H Quinn M Cipriano-Steffens T Jayaprakash M Koebnick E Randal F . Identifying actionable strategies: using consolidated framework for implementation research (CFIR)-informed interviews to evaluate the implementation of a multilevel intervention to improve colorectal cancer screening. Implement Sci Commun. (2021) 2:57. doi: 10.1186/s43058-021-00150-9, 34059156 PMC8167995

[13] Kirk MA Kelley C Yankey N Birken SA Abadie B Damschroder L. A systematic review of the use of the consolidated framework for implementation research. Implement Sci. (2015) 11:72. doi: 10.1186/s13012-016-0437-z, 27189233 PMC4869309

[14] Brewer LC Williams DR. We’ve come this far by faith: the role of the black church in public health. Am J Public Health. (2019) 109:385–6. doi: 10.2105/AJPH.2018.304939, 30726121 PMC6366503

[15] Jones SC Schlundt D Williams N Smalls M Idrizi K Alexander LR . Challenges in disseminating evidence-based health promotion programs in faith community settings: what we need to include. Health Promot Pract. (2025) 26:579–91. doi: 10.1177/15248399241259688, 39066625 PMC11979308

[16] Emmons KM Mendez S Lee RM Erani D Mascioli L Abreu M . Data sharing in the context of community-engaged research partnerships. Soc Sci Med. (2023) 325:115895. doi: 10.1016/j.socscimed.2023.115895, 37062144 PMC10308954

[17] Schmitt M Hawkins M Florsheim P. Key determinants in implementation processes: a systematic review using the consolidated framework for implementation research (CFIR). Implement Sci Commun. (2015) 6:89. doi: 10.1186/s43058-025-00712-1, 40846990 PMC12374266

[18] Damschroder LJ Aron DC Keith RE Kirsh SR Alexander JA Lowery JC. Fostering implementation of health services research findings into practice: a consolidated framework for advancing implementation science. Implement Sci. (2009) 4:50. doi: 10.1186/1748-5908-4-50, 19664226 PMC2736161

[19] Waltz TJ Powell BJ Fernández ME Abadie B Damschroder LJ. Choosing implementation strategies to address contextual barriers: diversity in recommendations and future directions. Implement Sci. (2019) 14:42. doi: 10.1186/s13012-019-0890-9 31036028 PMC6489173

[20] Powell BJ Waltz TJ Chinman MJ Damschroder LJ Smith JL Matthieu MM . A refined compilation of implementation strategies: results from the expert recommendations for implementing change (ERIC) project. Implement Sci. (2015) 10:21. doi: 10.1186/s13012-015-0209-1, 25889199 PMC4328074

[21] Waltz TJ Schlundt D Jones S Gishe J Alexander L Williams N . Development of an adapted glossary of implementation strategies for supporting behavioral health program uptake in faith-based communities. Implement Sci Commun. (2026) 7:56. doi: 10.1186/s43058-026-00878-2, 41731585 PMC13036926

[22] Holt CL Shelton RC Allen JD Bowie J Jandorf L Santos SLZ . Development of tailored feedback reports on organizational capacity for health promotion in African American churches. Eval Program Plann. (2018) 70:99–106. doi: 10.1016/j.evalprogplan.2018.07.002 30041105 PMC6077099

[23] Haughton J Takemoto ML Schneider J Hooker SP Rabin B Brownson RC . Identifying barriers, facilitators, and implementation strategies for a faith-based physical activity program. Implement Sci Commun. (2020) 1:51. doi: 10.1186/s43058-020-00043-3, 32885207 PMC7427873

[24] Idnay B Zhang Y Therese KS Nestor JG Chung WK Weng C. Returning aggregate research results to participants: a scoping review of current practices, preferences and challenges. BMJ Open. (2025) 15:e107270. doi: 10.1136/bmjopen-2025-107270, 41320209 PMC12666177

